# Correlation Between Hemoglobin Levels and Severity of Coronary Artery Disease in Patients With Myocardial Infarction

**DOI:** 10.7759/cureus.99929

**Published:** 2025-12-23

**Authors:** Muhammad Uzair Khalid, Ali Bajwa, Muhammad Yasir, Hamza Javed, Muhammad Bilal Zahid, Arshbeer Singh Sandhu, Babar Soomro, Somia Bibi, Mudassar Nisar, Zoya Usman, Menahil Khalid

**Affiliations:** 1 Internal Medicine, Jinnah Hospital, Lahore, PAK; 2 Surgery, Spire Cardiff Hospital, Cardiff, GBR; 3 Acute Medicine, Royal Derby Hospital, Derby, GBR; 4 Internal Medicine, Diana, Princess of Wales Hospital, Grimsby, GBR; 5 Internal Medicine, Shalamar Medical and Dental College, Lahore, PAK; 6 Cardiology, Jinnah Hospital, Lahore, PAK; 7 Neurosciences, University Hospital Coventry and Warwickshire, Coventry, GBR; 8 Cardiothoracic Surgery, St Bartholomew’s Hospital, London, GBR; 9 Internal Medicine, Rawalpindi Medical University, Rawalpindi, PAK; 10 Internal Medicine, George Eliot Hospital, Nuneaton, GBR; 11 Internal Medicine, Rashid Latif Medical College, Lahore, PAK

**Keywords:** coronary artery disease, correlation, disease severity, hemoglobin level, myocardial infarction

## Abstract

Background

Coronary artery disease (CAD) is one of the major causes of mortality around the globe. This study aimed to determine the relationship between hemoglobin (Hb) levels and CAD severity in patients with myocardial infarction (MI).

Methodology

This retrospective study was conducted among 265 MI patients at Jinnah Hospital’s Cardiology Unit, Lahore, Pakistan, between March 2022 and March 2023. Consecutive sampling and predefined criteria guided patient enrollment. A predefined proforma collected patient data from the medical record, which was then categorized into non-severe CAD and severe CAD groups based on Gensini scores. Independent t-tests and chi-square tests were used to compare variables between the groups. Pearson’s correlation coefficient determined the Hb level and CAD severity relationship, while linear regression analysis assessed the Hb level’s predictive value for CAD severity. A p-value <0.05 indicated statistical significance. Data analysis was performed using SPSS software, version 25 (Released 2017; IBM Corp., Armonk, NY, USA).

Results

Of 265 patients, 170 (64.15%) had non-severe CAD and 95 (35.85%) had severe CAD. The frequency of anemia was also high (n = 142, 53.58%) in the study population. Patients with severe CAD had significantly lower Hb levels (10.12 ± 3.42 g/dL) compared to those with non-severe CAD (12.89 ± 1.90 g/dL). Significant differences were noted in Gensini scores (p < 0.001) and Hb levels (p < 0.001) between the study groups. A strong negative correlation was noted between Hb levels and CAD severity (r = -0.72, 95% confidence interval (CI) = -0.76 to -0.70, p = 0.001). Regression analysis validated Hb level as a significant determinant of CAD severity (negative unstandardized coefficient = -3.10, standardized coefficient = -0.72, 95% CI = -4.90 to -1.50, p = 0.001, R² = 0.46).

Conclusions

This study presented a significant inverse association between Hb levels and CAD severity in MI patients. Lower Hb levels were correlated with more severe CAD, suggesting its potential as a risk marker and clinical instrument for identifying high-risk patients, facilitating prompt interventions, and guiding therapy.

## Introduction

Coronary artery disease (CAD) is a major global health concern, ranking among the top causes of illness and death worldwide. CAD develops when the coronary arteries become narrow or blocked due to atherosclerosis, leading to decreased blood flow to the heart muscle and potentially causing symptoms such as chest pain, dyspnea, and sweating [1,2]. CAD can present in various ways, including acute coronary syndrome (ACS) and chronic coronary syndrome. ACS comprises three main subtypes, namely, non-ST-segment elevation myocardial infarction (NSTEMI), unstable angina, and ST-segment elevation myocardial infarction (STEMI). NSTEMI and STEMI represent acute myocardial infarctions (MIs), a grave and potentially lethal manifestation of ACS, characterized by sudden and severe myocardial ischemia [3].

The global burden of CAD is rising in both developed and developing countries [4]. The World Health Organization (WHO) reports that cardiovascular disorders are responsible for 17.9 million deaths annually, representing approximately 31% of global fatalities [5,6]. In Pakistan, the prevalence of CAD is approximately 18.90%, with a substantial proportion of patients experiencing acute MI [5,7].

Beyond the traditional risk factors for CAD, including hypertension, diabetes mellitus, familial cardiovascular disease history, dyslipidemia, obesity, tobacco smoking, unhealthy dietary habits, and sedentary behavior, recent studies suggest that hemoglobin concentrations may substantially influence CAD pathogenesis and exacerbate disease severity [8-10]. Considering the escalating burden of CAD, comprehensive research is crucial to uncover novel risk factors that could serve as therapeutic targets for disease management and prevention.

Anemia is defined as hemoglobin (Hb) levels of less than 13.0 g/dL and 12.0 g/dL in males and females, respectively. It is a common comorbidity in patients with CAD [11,12]. Emerging evidence suggests low Hb levels may play a significant role in the development and progression of CAD, potentially through mechanisms such as tissue hypoxia, increased cardiac output, left ventricular hypertrophy, inflammation and oxidative stress, and endothelial dysfunction. Tissue hypoxia occurs when reduced oxygen delivery to the myocardium exacerbates ischemia and worsens cardiac function. Increased cardiac output is a compensatory mechanism where the heart attempts to compensate for reduced oxygen-carrying capacity by increasing cardiac output, leading to increased myocardial oxygen demand and further strain on the heart. Left ventricular hypertrophy is a chronic complication of anemia, which can increase the risk of heart failure and arrhythmias. Inflammation and oxidative stress contribute to a pro-inflammatory state, promoting atherosclerosis and CAD progression. Endothelial dysfunction impairs nitric oxide production and increases vascular tone, worsening CAD [12-14].

In Pakistan, anemia is very common and affects a large population [15]. The relationship between Hb levels and CAD severity has been explored in several studies globally, with most reporting an inverse correlation between the two variables. This correlation suggests that Hb levels may be a useful marker for identifying patients at high risk of CAD and a potential target for therapeutic intervention. However, the link between Hb levels and CAD severity in MI patients is not well established, especially in resource-constrained environments common in developing nations [16-20].

This study aimed to determine the correlation between Hb levels and CAD severity in patients with MI in Pakistan, as research on this topic in Pakistan is limited. Assessment of this association could lead to improved understanding and management of CAD. The study findings could also guide the development of targeted interventions to reduce CAD-related morbidity and mortality, particularly in resource-constrained countries.

## Materials and methods

Study design and study population

This retrospective analysis was performed at the Department of Cardiology, Jinnah Hospital, Lahore, Pakistan, between March 2022 and March 2023. Of the 375 patients who were admitted with acute MI during the study period, we enrolled only 265 patients with acute MI who had complete coronary angiography records, selected via consecutive sampling based on specific eligibility criteria. The minimum sample size was determined (n = 245) using the OpenEpi calculator, assuming a 17.50% previous prevalence of coronary heart disease, a 95% confidence level, a 5% margin of error, and an 80% study power [21]. Institutional ethics approval and informed consent from all participants were obtained before the study.

Inclusion and exclusion criteria

This study enrolled patients of both sexes, aged ≥18 years, with a verified diagnosis of ACS based on ECGs, cardiac biomarkers, coronary angiography, and complete medical records. Exclusion criteria included patients with a history of cardiac conditions (previous episode of MI, pre-existing bundle branch block, non-sinus rhythm on ECG, congenital heart defects, and heart failure) or prior cardiac interventions, polycythemia (Hb level of >16.5 g/dL for men and >16.0 g/dL for women), history of anticoagulation therapy, recent blood transfusion (<3 months), pregnancy, chronic kidney or liver disease, immunodeficiency disorders, active infections (with raised total white blood cell count, C-reactive protein, or erythrocyte sedimentation rate), malignancy, and recent surgical procedures (within four months). By excluding these patients, we aimed to minimize confounding factors and attribute observed changes to ACS rather than underlying comorbidities.

Primary and secondary objectives

This study’s principal aim was to determine the association between Hb levels and CAD severity, as quantified by the Gensini score. Additional objectives included contrasting Hb levels in patients with severe CAD versus non-severe CAD and assessing the utility of Hb level as a predictor of CAD severity.

Evaluation of study variables

Using the cyanmethemoglobin method, Hb levels were measured from blood samples as per hospital protocols following the guidelines of the American College of Pathologists. Anemia is defined as Hb levels of less than 13.0 g/dL and 12.0 g/dL in males and females, respectively [14]. MI diagnosis was based on the American Heart Association criteria, including prolonged chest pain (≥30 minutes), characteristic ECG changes, and elevated cardiac enzymes [10]. CAD was diagnosed via coronary angiography, with significant stenosis (>50% vessel diameter) considered diagnostic. CAD severity was assessed using the validated Gensini scoring system, which calculates scores based on stenosis severity and lesion location. Patients were categorized as having non-severe CAD (Gensini score ≤50) or severe CAD (Gensini score >50) [6,10]. Two experienced cardiologists, blinded to patient outcomes, independently scored angiograms. Inter-rater reliability was assessed using the intraclass correlation coefficient (0.85), indicating good agreement.

Data collection

Data were collected from medical records using a customized proforma. The proforma used standardized data handling, administered by trained researchers, with double-entry verification to ensure accuracy. It had two sections, A and B. Section A captured demographic and clinical data, including age, gender, and the presence or absence of conventional CAD risk factors, such as family history, hypertension, diabetes mellitus, dyslipidemia, and smoking history. Section B recorded the results of diagnostic tests, including serum Hb levels, lipid profiles, cardiac biomarkers, ECG findings, and coronary angiography results, all performed at Jinnah Hospital as part of routine patient management.

Data analysis

Statistical analysis was performed using SPSS Statistics for Windows, Version 25 (IBM Corp., Armonk, NY, USA). Categorical variables were summarized using descriptive statistics, including frequencies and percentages, while continuous variables were expressed as mean ± SD. Due to the normal distribution of the data, as confirmed by the Shapiro-Wilk test (p > 0.05), parametric tests were applied. The independent t-test and chi-square test were used to compare quantitative and qualitative variables between the groups. Pearson’s correlation coefficient was used to evaluate the association between Gensini scores and Hb levels. Furthermore, linear regression analysis was performed to assess the predictive value of Hb levels on Gensini scores. A p-value <0.05 was deemed statistically significant.

## Results

Among 265 patients, 170 (64.15%) were diagnosed with non-severe CAD, whereas 95 (35.85%) had severe CAD. In the study population, 142 (53.58%) had anemia, while 123 (46.42%) had normal Hb levels. The average values (±SD) for key factors were as follows: age: 59.81 ± 17.90 years, Gensini score: 49.86 ± 34.75, and Hb levels: 12.18 ± 4.60 g/dL.

Table 1 presents the study population’s demographic and clinical characteristics, showing significant variation between the non-severe CAD and severe CAD groups in crucial parameters such as Gensini score and Hb levels (p < 0.001). Additionally, severe CAD was more prevalent among patients with specific predisposing factors, including higher age, male sex, familial history of CAD, hypertension, and diabetes mellitus. However, no statistically significant variations were observed in the distribution of these contributing variables between the two study groups (p > 0.05).

**Table 1:** Demographic and clinical features of the study group. The table shows test statistics and p-values for two tests, marked as follows: ^*^ for independent t-tests and ^+^ for chi-square tests, applying to both test statistic values and p-values. CAD: coronary artery disease; N: study population size; n: sample size for each group or category; %: percentage; SD: standard deviation

Variables, N = 265		Expression of variables	Study groups as per CAD severity		Independent t-test/Chi-square test	
					Test statistics	
			Non-severe CAD group, n = 170 (64.15%)	Severe CAD Group, n =95 (35.85%)	t-value for independent-test/χ²-value for chi-square test	P-values
Age (years) (mean ± SD)		59.81 ± 17.90	58.60 ± 15.22	60.45 ± 18.08	0.86^*^	0.14^*^
Gensini score (mean ± SD)		49.86 ± 34.75	27.50 ± 15.24	73.56 ± 36.29	12.93^*^	<0.001^*^
Hemoglobin level (g/dL) (mean ± SD)		12.18 ± 4.60	12.89 ± 1.90	10.12 ± 3.42	15.70^*^	<0.001^*^
Gender	Male, n (%)	164 (61.88)	106 (62.35)	58 (61.05)	0.35^+^	0.80^+^
	Female, n (%)	101 (38.12)	64 (37.65)	37 (38.95)		
Family history of CAD	Yes, n (%)	82 (30.94)	34 (20.00)	48 (50.52)	2.32^+^	0.07^+^
	No, n (%)	183 (69.06)	136 (80.00)	47 (49.48)		
Hypertension	Yes, n (%)	178 (67.16)	112 (65.88)	66 (69.47)	0.46^+^	0.80^+^
	No, n (%)	87 (32.84)	58 (34.12)	29 (30.53)		
Diabetes mellitus	Yes, n (%)	150 (56.60)	102 (60.00)	48 (50.52)	0.32^+^	0.50^+^
	No, n (%)	115 (43.40)	68 (40.00)	47 (49.48)		
Dyslipidemia	Yes, n (%)	110 (41.51)	70 (41.17)	40 (42.10)	0.20^+^	0.90^+^
	No, n (%)	155 (58.49)	100 (58.83)	55 (57.90)		
Smoking status	Yes, n (%)	112 (42.27)	73 (42.94)	39 (41.10)	4.80^+^	0.10^+^
	No, n (%)	153 (57.73)	97 (57.06)	56 (58.90)		

Table 2 demonstrates a significant inverse association between Hb levels and the severity of CAD, as determined by Pearson’s correlation analysis. This linkage indicates that higher Gensini scores were accompanied by diminished Hb levels, signifying a negative relationship between CAD severity and Hb levels.

**Table 2:** Correlation between hemoglobin levels and coronary artery disease severity. CAD: coronary artery disease; CI: confidence interval

Variables of patients with myocardial infarction, N = 265	Study groups based on CAD severity		Independent t-test		Pearson’s correlation		
			Test statistics		Test statistics		
	Non-severe CAD group	Severe CAD group	t-value	P-value	Correlation coefficient (r)	95% CI	P-value
Gensini score	27.50 ± 15.24	73.56 ± 36.29	12.93	<0.001	-0.72	-0.76 to -0.70	0.001
Hemoglobin level (g/dL) (mean ± SD)	12.89 ± 1.90	10.12 ± 3.42	15.70	<0.001			

Table 3 shows that the linear regression analysis yielded a strong predictive model, with an R² value of 0.46 and a highly significant p-value (p = 0.001). This indicates a substantial inverse relationship between Hb levels and Gensini scores. Specifically, the negative regression coefficient suggests that lower Hb levels were closely tied to higher Gensini scores, indicating more severe CAD.

**Table 3:** Analysis of the correlation between hemoglobin levels and CAD severity using a linear regression model. CAD: coronary artery disease; CI: confidence interval

Variable	Test statistics for the simple linear regression model					
	Unstandardized coefficient	Standardized coefficient	95% CI	P-value	R^2 ^value	P-value of the F test
Hemoglobin level	-3.10	-0.72	-4.90 to -1.50	0.001	0.46 (46.00%)	0.001

## Discussion

CAD is a leading cause of mortality globally. Early diagnosis, risk stratification, and intervention can substantially reduce CAD-related mortality. While traditional risk factors such as hypertension, diabetes, obesity, sedentary lifestyle, family history, and smoking are well-established, emerging evidence suggests a significant association between low Hb levels and CAD [1]. Understanding the relationship between Hb levels and CAD severity can enhance patient care and outcomes, especially in resource-limited settings. This study explores the relationship between Hb levels and CAD severity, determining the prevalence of risk factors and their distribution between non-severe and severe CAD patient groups.

Among the 265 patients in our study, 170 (64.15%) had non-severe CAD, whereas 95 (35.85%) had severe CAD. Another study from the Pakistani population presented similar findings about the distribution of CAD based on its severity [6]. In contrast, an Indian study found a higher proportion of severe CAD in specific populations. The discrepancy in CAD severity between studies may be attributed to differences in the prevalence of traditional cardiovascular risk factors among the patient populations [22]. Regarding the Hb levels among the study population, anemia was present in 142 (53.58%) individuals, whereas 123 (46.42%) had normal Hb levels. Another study reported a similar prevalence of anemia in an identical population [11].

Assessment of the demographic features of the study population indicated that participants with severe CAD had a higher mean age (60.45 ± 18.08 years) than patients with non-severe CAD (58.60 ± 15.22 years). Furthermore, CAD was more prevalent among male patients, accounting for 164 (61.88%) cases. These findings are in line with previous research that reported comparable demographic trends in CAD patients [10].

Notably, hypertension was the most common established risk factor in the study population, followed by diabetes mellitus, smoking, dyslipidemia, and family history of CAD. A similar distribution of predisposing factors has been noted in previous studies [6-9,11].

The findings of the present study demonstrate that lower Hb levels are strongly associated with more severe CAD, as reflected by higher Gensini scores. This correlation is consistent with previous global research highlighting that low Hb level is associated with CAD severity. A Chinese study demonstrated that Hb levels have a significant role in the development and progression of CAD [1]. A study from a similar population also highlighted the association between lower Hb levels and increased risk of CAD [11]. A study from the United States reported consistent findings, showing that decreased Hb levels lead to increased CAD risk and severity [13]. Another study from Saudi Arabia also validated that anemia increased mortality among cardiac patients [14]. In a 25-year follow-up study among the general Japanese population, low Hb level increased the incidence and severity of CAD [16]. German studies have also supported the significant role of Hb levels in the progression of cardiovascular disease severity [17,18]. The results of this study support a statistically significant inverse association between Hb levels and CAD severity in MI patients, consistent with previous research findings. However, due to the retrospective and observational design, we cannot infer causality.

In the literature, it has been described that the pathophysiological mechanisms underlying this association between Hb levels and CAD development and progression are multifaceted. Low Hb levels lead to tissue hypoxia, which exacerbates ischemia and worsens cardiac function. Increased cardiac output is a compensatory mechanism where the heart attempts to compensate for reduced oxygen-carrying capacity by increasing cardiac output, leading to increased myocardial oxygen demand and further strain on the heart. Left ventricular hypertrophy is a chronic complication of anemia, which can increase the risk of heart failure and arrhythmias. Inflammation and oxidative stress contribute to a pro-inflammatory state, promoting atherosclerosis and CAD progression. Endothelial dysfunction impairs nitric oxide production and increases vascular tone, worsening CAD [12-14,16,17].

The clinical implications of this study suggest that Hb levels can serve as a biomarker and risk factor for CAD severity, guiding treatment strategies and risk assessment after confirmation by future studies with different study designs. Measuring Hb levels can identify high-risk patients and inform personalized treatment plans, potentially improving cardiac health and reducing CAD burden. Integrating the assessment and management of Hb levels into clinical practice may improve patient outcomes, emphasizing the need for increased prevention strategies, especially in developing countries.

Although this study’s findings contribute to the growing body of evidence supporting the use of Hb levels as a predictor for CAD severity assessment, it has limitations, including its retrospective design, single-center setting, and limited sample size. Additionally, the use of simple linear regression, no adjustment for potential confounding variables, such as single-time-point Hb measurement, and no assessment of nutritional and socioeconomic status of the patients may have influenced the results. Future research should address these limitations and focus on longitudinal, interventional, and multi-center studies to establish causality, determine the effect of Hb level management, and enhance generalizability. Integrating Hb levels into existing CAD risk scores may improve predictive accuracy and clinical utility.

## Conclusions

This study showed an inverse correlation between Hb levels and CAD severity in MI patients. Lower Hb levels were associated with more severe CAD, highlighting its potential as a risk indicator and diagnostic tool. Given that measuring Hb levels is easy and cost-effective, it can be used for cardiac risk assessment, especially in resource-limited settings. Timely intervention in cardiac patients with low Hb levels may improve outcomes and reduce mortality. Overall, this study only supports the association of Hb levels with CAD severity due to its retrospective design. Therefore, further studies with different study designs are needed to confirm the role of Hb in assessing CAD severity and guiding treatment.

## Appendices

Research proforma 

**Table 4:** Section A of the self-designed research proforma covering the demographic and clinical data.

Serial number	Questions	Response options
1.	What was the age of the patient?	In years
2.	What was the gender of the patient?	a) Male; b) Female
3.	What were the presenting complaints of the patient?	Description
4.	What was the duration of presenting complaints?	In Minutes
5.	Presence of family history of coronary artery disease?	a) Yes; b) No
6.	Past history of hypertension?	a) Yes; b) No
7.	Past history of diabetes mellitus?	a) Yes; b) No
8.	Past history of dyslipidemia?	a) Yes; b) No
9.	Past history of smoking?	a) Yes; b) No
10.	Past history of coronary artery disease/cardiac, or any other surgery?	a) Yes; b) No
11.	Past treatment history?	a) Yes; b) No
12.	Presence of any chronic disease other than those mentioned above?	a) Yes; b) No
13.	What were the physical examination observations of the patient? (vitals, general, and systemic)	Description
14.	What were the findings of the past medical record?	Description

**Table 5:** Section B of the self-designed research proforma covering the results of diagnostic tests.

Serial number	Questions	Response options
1.	What were the electrocardiogram findings?	Description
2.	What was the cardiac biomarker (troponin I) level?	In ng/mL
3.	What were the coronary angiography findings?	Description
4.	What was the Gensini score?	Numerical value
5.	What was the severity of coronary artery disease based on the Gensini score?	a) Non-severe coronary artery disease group (up to 50); b) Severe coronary artery disease group (above 50)
6.	What was the hemoglobin level? (Anemia is defined as Hb levels of less than 13.0 g/dL and 12.0 g/dL in males and females, respectively)	In g/dL
7.	What was the status of hemoglobin levels?	a) Normal; b) Low hemoglobin levels (anemia)
8.	What was the serum lipid level?	In mg/dL
9.	What was the status of lipid levels?	a) Normal (less than 200 mg/dL); b) Abnormal (above 200 mg/dL)
10.	Was there any abnormality in the liver function tests?	a) Yes; b) No
11.	Was there any abnormality in the renal function tests?	a) Yes; b) No
12.	Was the C-reactive protein value within the normal range?	a) Yes; b) No
13.	Was the erythrocyte sedimentation rate value within the normal range?	a) Yes; b) No
14.	Was the white blood cell count value within the normal range?	a) Yes; b) No
